# Enhancing infectious intestinal disease diagnosis through metagenomic and metatranscriptomic sequencing of 1000 human diarrhoeal samples

**DOI:** 10.1186/s13073-025-01478-w

**Published:** 2025-05-20

**Authors:** Edward Cunningham-Oakes, Blanca M. Perez-Sepulveda, Yan Li, Jay C. D. Hinton, Charlotte A. Nelson, K. Marie McIntyre, Maya Wardeh, Sam Haldenby, Richard Gregory, Miren Iturriza-Gómara, Christiane Hertz-Fowler, Sarah J. O’Brien, Nigel A. Cunliffe, Alistair C. Darby, on behalf of the INTEGRATE consortium, Charlotte A. Nelson, K. Marie McIntyre, Maya Wardeh, Sam Haldenby, Miren Iturriza-Gómara, Christiane Hertz-Fowler, Sarah J. O’Brien, Nigel A. Cunliffe, Frederick J Bolton, Rob M Christley, Helen E Clough, Susan Dawson, Elizabeth Deja, Ann E Durie, Neil Hall, Debbie Howarth, Lirije Hyseni, Kathryn Jackson, Lucy Jones, Trevor Jones, Lois Orton, Jane A Pulman, Alan D Radford, Danielle Reaves, Helen K Ruddock, Darlene A Snape, Debbi Stanistreet, Tamara Thiele, David Williams, Craig Winstanley, Kate Dodd, Peter J Diggle, Alison C Hale, Barry S Rowlingson, Jim Anson, Caroline Corless, Viki Owen, Malcolm Bennett, Lorraine Bolton, John Cheesbrough, Katherine Gray, David Orr, Lorna Wilson, Andrew R Dodgson, Ashley McEwan, Paul Cleary, Alex J Elliot, Ken H Lamden, Lorraine Lighton, Catherine M McCann, Matthieu Pegorie, Nicola Schinaia, Anjila Shah, Gillian E Smith, Roberto Vivancos, Bernard Wood, Rikesh Bhatt, Dyfrig A Hughes, Rob Davie, Simon de Lusigna, Filipa Ferreira, Mariya Hriskova, Sam O’Sullivan, Stacy Shinneman, Ivelina Yonova

**Affiliations:** 1 Institute of Infection, University of Liverpool, Veterinary & Ecological Sciences, Liverpool, UK; 2 NIHR Health Protection Research Unit in Gastrointestinal Infections, Liverpool, UK; 3 Department of Clinical Infection, Microbiology and Immunology, Institute of Infection, Veterinary and Ecological Sciences, University of Liverpool, Liverpool, UK; 4 Centre for Genomic Research, Institute of Systems, Molecular and Integrative Biology, University of Liverpool, Liverpool, UK; 5 School of Natural and Environmental Sciences, Newcastle University, Newcastle, UK; 6 Department of Computer Science, University of Liverpool, Liverpool, UK; 7 Department of Livestock and One Health, Institute of Infection, Veterinary and Ecological Sciences, University of Liverpool, Liverpool, UK; 8 Department of Mathematics, University of Liverpool, Liverpool, UK; 9 GSK Vaccines for Global Health Institute, Siena, Italy

**Keywords:** Microbiome, Culture-independent, Metagenome, Metatranscriptome, Diagnostics, Genomics, Pathogens

## Abstract

**Background:**

Current surveillance of diarrhoeal disease is hindered by limitations of traditional diagnostic approaches, which often fail to identify the causative organism, particularly for novel or hard-to-culture bacterial pathogens. Sequencing nucleic acids directly from stool can overcome such constraints, but such approaches need to reliably detect pathogens identifiable by conventional methods.

**Methods:**

As part of the INTEGRATE study, we analysed stool microbiomes from 1067 patients with gastroenteritis symptoms using direct sequencing, and compared findings with standard diagnostic techniques (culture, immunoassay, microscopy, and single-target PCR) and molecular assays (Luminex xTAG GPP) for detection of bacterial and viral pathogens in the UK.

**Results:**

We found strong positive correlations between metatranscriptomic reads and traditional diagnostics for six out of 15 pathogens. The metatranscriptomic data were highly correlated with the Luminex assay for eight out of 14 pathogens. In contrast, metagenomic sequencing only showed a strong positive correlation with traditional diagnostics for three of 15 pathogens, and with Luminex for four of 14 pathogens. Compared with metagenomics, metatranscriptomics had increased sensitivity of detection for four pathogens, while metagenomics was more effective for detecting five pathogens.

Metatranscriptomics gave near-complete transcriptome coverage for Human mastadenovirus F and detected *Cryptosporidium* via identification of *Cryptosporidium parvum* virus (CSpV1). A comprehensive transcriptomic profile of *Salmonella enterica* serovar Enteritidis was recovered from the stool of a patient with a laboratory-confirmed *Salmonella* infection. Furthermore, comparison of RNA/DNA ratios between pathogen-positive and pathogen-negative samples demonstrated that metatranscriptomics can distinguish pathogen-positive/negative samples and provide insights into pathogen biology. Higher RNA/DNA ratios were observed in samples that tested positive via gold-standard diagnostics.

**Conclusions:**

This study highlights the power of directly sequencing nucleic acids from human samples to augment gastrointestinal pathogen surveillance and clinical diagnostics. Metatranscriptomics was most effective for identifying a wide range of pathogens and showed superior sensitivity. We propose that metatranscriptomics should be considered for future diagnosis and surveillance of gastrointestinal pathogens. We assembled a rich data resource of paired metagenomic and metatranscriptomic datasets, direct from patient stool samples, and have made these data publicly available to enhance the understanding of pathogens associated with infectious intestinal diseases.

**Supplementary Information:**

The online version contains supplementary material available at 10.1186/s13073-025-01478-w.

## Background

The incidence of infectious intestinal disease (or acute gastroenteritis) is estimated to be 18 million cases each year in the United Kingdom (UK) [1]. About 25% of infected people experience diarrhoeal and related gastrointestinal symptoms. The current mainstay for identifying gastrointestinal pathogens in faecal specimens in the UK is conventional laboratory techniques, including microscopy and antigen detection, and increasingly, molecular assays such as nucleic acid amplification [2].

Although conventional and polymerase chain reaction (PCR)-based approaches (such as BioFire Panels) are validated for clinical laboratory use [2], both focus on a single gene or set of characteristics, providing limited information about pathogens [3]. In the case of bacterial culture, the time required for growth, lack of sensitivity, and the challenge of culturing fastidious organisms cause diagnostic delays [3]. Current methods lack the sensitivity required to detect pathogens that are present intermittently or in low numbers [4]. In contrast, PCR-based methods use target sequences for organism detection, resulting in increased sensitivity and no strict requirement for the prior growth of organisms [3]. This approach has advanced diagnostics for viruses [5] and parasites [6], where isolation from stool is slower and more complex than for bacteria.

Whilst PCR-based methods are more sensitive than conventional (traditional) methods, PCR-based, both Methods are limited by their targeted approach [7] and cannot achieve the strain-level discrimination required for outbreak monitoring [8]. Inevitably, molecular assays target known genes from well-characterised organisms [7], meaning that unexpected pathogens and unique genes will be missed. Whole-genome sequencing partly overcomes the need for curated gene targets, but still requires either enrichment or capture of the target pathogen.

The speed and sensitivity of metagenomic and metatranscriptomic data analysis [9] has been significantly enhanced by *k*-mer-based methods, an approach that has been widely adopted in many popular workflows [10, 11] to identify pathogens in metagenomic samples through database matching. The computational efficiency of *k*-mers is ideal for high-throughput sequencing applications [12]. However, it is important to note that sequencing errors and the comprehensiveness of the databases used [13] can influence the effectiveness of *k*-mer-based approaches.

Metagenomic and metatranscriptomic sequencing of clinical samples have been proposed as valuable approaches for the future of pathogen detection [7]. Multi-omics approaches are increasingly used in various contexts, including disease subtyping [14], biomarker discovery [15], and functional profiling [16]. However, the systematic evaluation of multi-omic approaches within routine diagnostic frameworks, particularly for community-acquired gastrointestinal (GI) pathogens, remains limited. Given metagenomics and metatranscriptomics have shown promise in other settings [17], it is critical to benchmark the performance of both metagenomics and metatranscriptomics against established diagnostic pathways.

The INTEGRATE study [18] compared traditional diagnostic methods (culture, immunoassay, microscopy, and single-target PCR) with advanced molecular methods (Luminex xTAG GPP) and genome-based microbiological techniques for identifying community-acquired gastrointestinal pathogens [18]. Here, we present data generated by next-generation sequencing of the stool microbiomes of 1067 patients with symptoms of gastroenteritis, with the aim of systematically evaluating the diagnostic potential of metagenomic and metatranscriptomic sequencing by benchmarking their performance against gold-standard clinical laboratory diagnostics for GI pathogens. We considered the comparative benefits of different sequencing types in various scenarios (right test, right time, right patient).

We use these data to show that both metagenomic (DNA) and metatranscriptomic (RNA) sequencing directly from stool can detect the major community-associated GI pathogens in the United Kingdom. We found that metagenomic and metatranscriptomic sequencing have distinctive features for pathogen detection and discovered that metatranscriptomics offers unexpected benefits for pathogen surveillance.

All the data have been made publicly available (PRJEB62473) to provide a rich data source for researchers to foster a deeper understanding of the pathogens associated with infectious intestinal diseases.

## Methods

### Patient recruitment and sample collection

Recruitment and sample collection was described previously [18]. Briefly, stool was collected from 1,067 members of the public with symptoms of acute gastroenteritis via practices in the Royal College of General Practitioners Research and Surveillance Centre National Monitoring Network (RCGP RSC NMN). Patients meeting inclusion criteria were invited to submit a stool sample for microbiological analysis. Consent was obtained for this procedure, as stool sampling is usually only performed if a case is severe or persistent. Patients who provided a stool sample were then recruited into the study.

### Sample processing

Faecal samples were received by one of three clinical laboratories (Royal Liverpool and Broadgreen University Hospitals NHS Trust, Central Manchester University Hospitals NHS Foundation Trust, or Lancashire Teaching Hospitals NHS Foundation Trust), and divided into two aliquots. One part of the sample was processed using Traditional methods (culture, immunoassay, microscopy or single-target PCR—see Additional File 1) at each laboratory; the other was processed using a combined molecular multiplex real-time polymerase chain reaction (PCR) and target-specific hybridisation probe (Luminex xTAG Gastrointestinal Pathogen Panel, Luminex, I032 C0324), supplemented with targets for Enteroaggregative *Escherichia coli* and Sapovirus*.* Nucleic acid extraction from faeces was performed using QIASymphany and EasyMag automated nucleic acid extraction platforms. Further details can be found in the primary study protocol [18]. See Additional File 2 for all diagnostic results.

### Metagenomic and metatranscriptomic sequencing

Illumina fragment libraries from DNA were prepared using NEBNext Ultra DNA Library Prep Kits (E7370L) after treatment with RNase to remove any residual RNA. For RNA sequencing, total RNA was treated with DNase to eliminate genomic DNA contamination. For the generation of dual-indexed, strand-specific RNA-Seq libraries, total RNA was extracted from all clinical samples. The RNA underwent ribosomal RNA (rRNA) depletion using the NEBNext rRNA Depletion Kit (Bacteria; E7850X) to more accurately differentiate less abundant transcripts. Following rRNA depletion, the NEBNext Ultra Directional RNA Library Prep Kit for Illumina (E7760) was used to prepare the RNA-Seq libraries. This kit includes a reverse transcription (RT) step that converts RNA into complementary DNA (cDNA). For all libraries, paired-end, 150-bp sequencing was subsequently performed on an Illumina HiSeq 4000. The average number of filtered reads per sample was 34 million**.**

### Quality control for second-generation sequencing reads

Modules from the MetaWRAP [19] (v1.3.2) pipeline were used to standardise metagenome analysis. The pipeline was deployed in a dedicated Conda environment, using the “manual installation” guide (see https://github.com/bxlab/metaWRAP). All paired-end reads underwent quality control using the MetaWRAP “read_qc” module to remove low-quality, adapter, and human sequence reads. The T2 T consortium complete human genome (GCF_009914755.1) and human mitochondrial genome (NC_012920.1) were used as references for the removal of human reads. All quality-controlled reads were deposited in The European Nucleotide Archive [20]

### Assigning taxonomy to genomic DNA and RNA reads and assessing microbiome diversity

DNA and RNA reads were used for taxonomic assignments with Kraken2 [10] (v2.1.2), using a custom database, which included all RefSeq complete genomes and proteins for archaea, bacteria, fungi, viruses, plants, protozoa, as well as all complete RefSeq plasmid nucleotide and protein sequences, and a false-positive minimised version of the NCBI UniVec database.

A confidence threshold of 0.1 with no minimum read threshold was used for assignments, and reports were generated for downstream BIOM file generation. For DNA sequencing data, read counts assigned to taxonomies in each sample were then re-estimated using the average read length of that sample, using Bracken [21] (v2.0). Kraken-biom (v1.0.1) was then used to generate BIOM file in json format (Additional File 3), using initial Kraken reports for RNA samples, and Bracken reports for DNA samples. Biom (v2.1.6) was then used to assign tabulated metadata to this biom file.

### Visualisation and comparison of taxa of interest in RNA and DNA

A taxonomy table was generated from the BIOM file in R (v4.2.2) using Phyloseq [22] (v1.42.0) and MicrobiotaProcess [23] (v1.10.3). Read-assigned taxonomy counts were parsed from this table for any samples with both metagenomic (DNA) and metatranscriptomic data (*n* = 985). Counts were extracted for the following taxa: *Adenoviridae*, *Campylobacter*, *Clostridioides difficile* (*C. difficile*), *Cryptosporidium*, *Escherichia coli* (*E. coli*), Norovirus, Rotavirus, *Salmonella*, *Shigella*, Sapovirus, *Vibrio cholerae *(*V. cholerae*) and *Yersinia enterocolitica* (*Y. enterocolitica*). These taxa were chosen to reflect the pathogen panels used during this study. RNA virus (Astrovirus, Norovirus, Rotavirus, and Sapovirus) read counts could not be extracted for this part of the analysis, as visualisations relied on the presence of DNA reads. DNA and RNA counts were log-transformed and plotted against one another as a line graph using standard functions in ggplot2 [24] (v3.4.0). Visualisations were then used to assess the sensitivity of metagenomics and metatranscriptomics for the selected taxa, where we define sensitivity as the skew of data points towards either metagenomics (*x*-axis) or metatranscriptomics (*y*-axis). A 0,0 intercept line was included in each line graph to assist in illustrating sensitivity differences.

### Correlation of genomic reads assigned to taxa of interest with number of observed taxa and results from laboratory diagnostics

Associations between read counts and laboratory results (see Additional File 4) for organisms of interest were assessed using a multivariable linear regression model in MaAsLin 2 [25] (v1.6.0) under default settings. The introduction of another variable into the model (laboratory results) provided a point of reference. This allowed us to determine the relationship between any sample with sequencing data and laboratory results. As such, for this analysis, all sequenced patient samples (*n* = 1067) were used, even if they did not contain both metagenomic and metatranscriptomic data. Our approach allowed RNA virus read counts from metatranscriptomic data to be included in this analysis. To visualise the strength of correlations between laboratory results and pathogen-assigned sequencing reads, correlation coefficients and adjusted p-values from the model were tabulated and used to generate a heatmap (Additional File 4: Fig S1) with corrplot (v0.9.2). Correlations were considered statistically significant if the adjusted *p*-value (*q*-value) was below 0.25, following MaAsLin 2 usage recommendations [25]. Adjusted *p*-values were generated using the Benjamini–Hochberg Procedure.

### Comparative analysis of Adenovirus-associated *k*-mers in DNA and RNA

The extract_kraken_reads.py utility from Kraken-tools (v1.2) was used alongside Kraken2 reports to extract reads with *k*-mer profiles associated with the family *Adenoviridae* for samples that tested positive using either Traditional or Luminex methods. Samples where sequencing was not successful for both DNA and RNA were excluded from this analysis.

These reads were then mapped to the Human adenovirus F40 (Accession: NC_001454.1)) and F41 (DQ315364.2) genomes using HISAT2 [26] (v2.2.1) for splice-aware mapping. Coverage statistics for each subtype were calculated using samtools coverage and compiled into a single table (Additional File 5). For visualisation of whole genome coverage, BAM files were converted to BigWig format using deepTools [27] (v3.5.5), and visualised in IGV [28] (v.2.17.4).

### Identification of CSpV1 as a biomarker of Cryptosporidium infection

To understand why *Cryptosporidium*-associated *k*-mers showed a positive correlation with using gold-standard diagnostics in metatranscriptomic but not metagenomic sequencing data, we employed competitive mapping using PanGIA [29] (v1.0.0-RC6.2). We mapped quality-controlled reads from all INTEGRATE samples against a database containing representative and reference genomes of bacteria, archaea, and viruses in NCBI RefSeq (release 89). This helped to validate our *k*-mer-based results and offers a less computationally intensive alternative to mapping-based approaches for future users of *k*-mer-based databases. By aligning the reads to these genome sequences, we obtained a read count and depth of coverage for each organism. We then extracted entries associated with the term “*Cryptosporidium*” along with their corresponding scores and mapping information. PanGIA also accounts for many reads mapped equally well to other organisms and the percentage of identity of these hits and derived a confidence score from this, ranging from 0 to 1 for each query sequence at each taxonomy level. This allowed us to determine the certainty that the organism is truly present in the sequencing data. We then correlated RNA reads mapped CSpV1 with Traditional and Luminex diagnostic results for *Cryptosporidium*, using MaAsLin 2, as described in previous sections.

### Visualisation of a Salmonella transcriptome directly from stool

Metatranscriptomic reads from a sample of a patient with a later-confirmed (culture positive) *Salmonella* spp. infection underwent quality control, alignment, and quantification using the Bacpipe RNA-seq processing pipeline (v0.6.0). The GFF annotation [30] for the *Salmonella enterica* subsp. *enterica* serovar Enteritidis PT4 strain P125109 (Accession: GCA_015240635.1) was used in this analysis. Coverage tracks and annotation were visualised using JBrowse (v1.16.8). This visualisation can be found in https://s.hintonlab.com/study_74 [53].

#### Validating the complementary value of RNA and DNA by linking ratios to positive diagnoses

DNA and RNA read counts (READ_COUNT_RSNB, as generated by PanGIA) mapping to *Campylobacter*, *C. difficile* and *V. cholerae* were separately extracted for DNA and RNA reads. RNA/DNA ratios were calculated for each sample, and data were imported into R for analysis. In this analysis, *V. cholerae* served as the negative control, as this pathogen was not detectable using gold-standard diagnostics. Ratios were then tabulated, logged for visualisation purposes, and displayed as a violin plot with Wilcoxon test *p*-values. A threshold of *p* < 0.05 was used for significance. Plots were generated using the following R packages: ggplot2, ggpubr (v0.6.0), tidyverse (v2.0.0), patchwork (v1.3.0.9000) and svglite (v2.1.3).

## Results

### Metagenomics and metatranscriptomics show different levels of sensitivity for GI pathogens

**Fig. 1:**
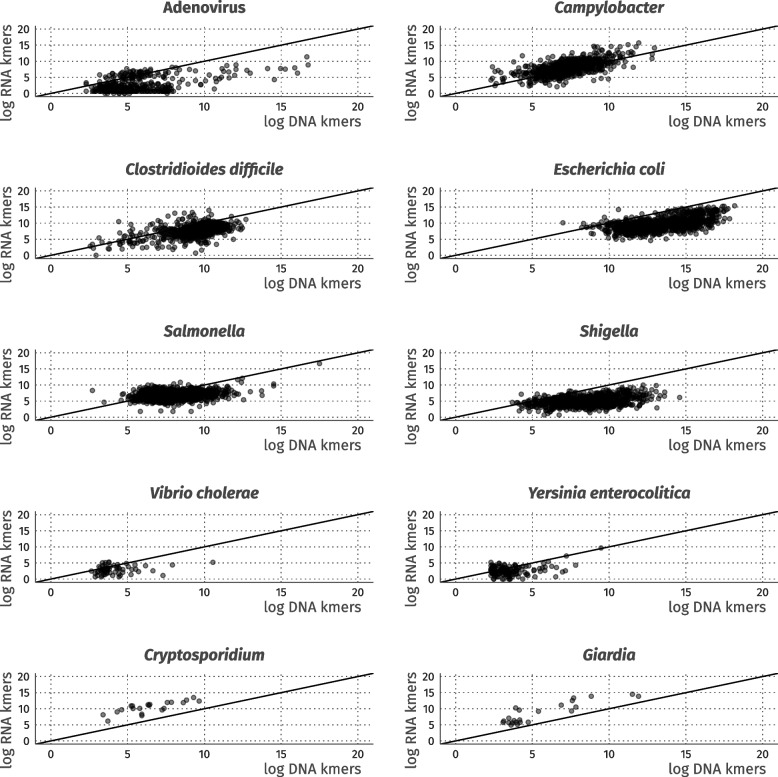
Visual overview and comparison of DNA (metagenomic) and RNA (metatranscriptomic) sequencing reads assigned to GI pathogens of relevance to the UK setting. For all graphs, the dashed (black) intercept line is provided to highlight the skew of sensitivity towards either DNA or RNA. This skew highlights the likelihood of identifying a pathogen in either DNA or RNA (e.g. Adenovirus can be detected more sensitively with DNA, whilst *Cryptosporidium* is detected more sensitively with RNA). Values shown are expressed as logarithmic units

The DNA and RNA extracted from a total of 1067 samples were sequenced, with 985 providing both metagenomic and metatranscriptomic data (see Additional File 3 for all *k*-mer counts and associated taxonomy from these samples). For *Campylobacter*, *Cryptosporidium* and *Giardia* (Fig. 1), metatranscriptomics showed greater sensitivity than metagenomics (see Methods for definition of sensitivity). In contrast, metagenomics displayed greater sensitivity than metatranscriptomics for the *Adenoviridae*, *Clostridium difficile*, pathogenic *Escherichia coli*, *Salmonella*, *Shigella* and *Yersinia enterocolitica* (Fig. 1). *Entamoeba histolytica* were not detected in either the metagenomic or metatranscriptomic datasets.

### The detection of GI pathogens in metagenomic and metatranscriptomic data is comparable to clinical laboratory results

**Fig. 2:**
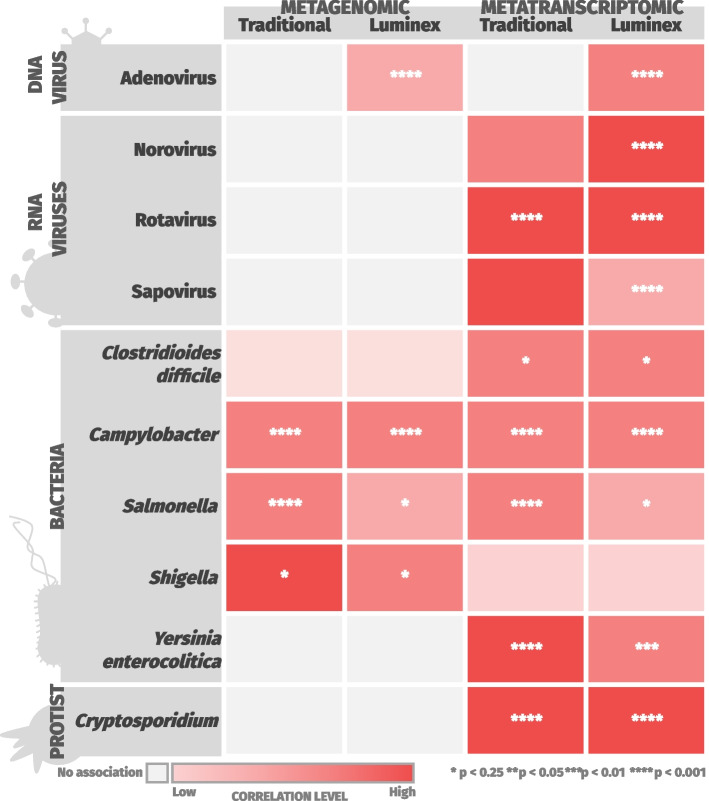
Statistically significant correlations were observed between sequencing data and laboratory tests for 10 out of 14 major GI community pathogens in the United Kingdom. Results where at least one statistically significant correlation was observed are shown. All correlations, whether significant or not, are displayed in Additional File 4: Fig S2. No statistically significant correlation was found between the sequencing and diagnostic test for Astrovirus, *E. histolytica*, *Giardia* or *V. cholera*. The darker the colour of a quadrant in a heatmap, the stronger the correlation (coefficient) between the detection of a pathogen in sequencing data (metagenomic or metatranscriptomic) and a laboratory result (Luminex or Traditional). Asterisks in quadrants indicate the statistical significance of correlations as follows: *: *p* < 0.25; **: *p* < 0.05; ***: *p* < 0.01; ****: *p* < 0.001

Our analysis showed that the pathogens detected in sequencing reads closely match results generated by laboratory diagnostics for Adenovirus, *C. difficile*, *Campylobacter*, *Cryptosporidium*, Norovirus, Rotavirus, *Salmonella*, Sapovirus, *Shigella* and *Y. enterocolitica* (Fig. 2; see Additional File 4: Fig S1 for a more complete overview of all laboratory diagnostic results). The total number of Traditional positives (*n* = 140) and Luminex positives (*n* = 485) are summarised in Table 1. Most major GI community pathogens in the UK were detected in both metagenomic and metatranscriptomic data, but RNA viruses could only be detected by metatranscriptomics. A summary of the “Traditional” methods used for pathogen diagnosis in the INTEGRATE study is presented in Table 2. A summary of all correlations between sequencing-based detection of viral, protist and bacterial pathogens in sequencing reads and laboratory data and their significance is provided in sections below, as well as Additional Files 4, 6 and 7.

**Table 1:** Summary of positive results by diagnostic method. This table summarises the number of positive pathogen diagnostic results undergoing comparison to sequencing in this study. The Luminex xTAG® Gastrointestinal Pathogen Panel (GPP) is a multiplexed nucleic acid test designed for the simultaneous qualitative detection and identification of multiple viral, bacterial, and parasitic nucleic acids in human stool specimens. Traditional methods encompass a variety of techniques based on routine diagnostic pathways employed in clinical laboratories, as detailed in Table 2

Pathogen	Traditional	Luminex
Adenovirus	7	7
*Campylobacter*	75	121
*Clostridioides difficile*	8	32
*Cryptosporidium*	19	33
*Entamoeba histolytica*	0	7
*Giardia*	8	51
Norovirus	2	84
Rotavirus A	1	20
*Salmonella*	15	44
Sapovirus	3	74
*Shigella*	2	11
*Vibrio cholerae*	0	0
*Yersinia enterocolitica*	0	1
**Total number of positive results**	140	485

**Table 2:** Summary of “Traditional” methods used at clinical laboratories during the INTEGRATE study. Samples were processed via routine diagnostic pathways at each laboratory involved in the study (see Additional File 1). Traditional assays for Enterotoxic and Enteroaggregative *E. coli*, as well as *E. coli* O157, were not available (only available in the Luminex xTAG GPP panel)

Test parameter	Liverpool	Manchester	Preston
Adenovirus 40/41	PCR	PCR	Immunoassay
Rotavirus A	PCR	PCR	Immunoassay
Norovirus GI/GII	PCR	PCR	Immunoassay and PCR
Sapovirus	PCR	PCR	Not available
*Clostridioides difficile* toxin A/B and GDH	Immunoassay	Immunoassay	Immunoassay
*Salmonella*	Culture	Culture	Culture
*Shigella*	Culture	Culture	Culture
*Campylobacter* (*C. jejuni*,* C. coli*,* C. lari*)	Culture	Culture	Culture
*E. coli* O157	Culture	Culture	Culture
Enterotoxigenic *E. coli* (ETEC) LT/ST	Not available	Not available	Not available
Enteroaggregative *E. coli*	Not available	Not available	Not available
*Yersinia enterocolitica*	Culture	Culture	Not available
*Vibrio cholerae*	Culture	Culture	Culture
Shigella-like toxin producing *E. coli* (STEC)	Not available	Not available	Not available
*Giardia lamblia*	Microscopy	Immunoassay	Immunoassay
*Cryptosporidium*	Microscopy	Immunoassay	Immunoassay
*Entamoeba histolytica*	Microscopy	Microscopy	Microscopy

### Viral pathogens

DNA viruses such as Adenovirus were detected in both the metagenomic and metatranscriptomic datasets. For Adenovirus, positive correlations were observed between detection in metagenomic reads, metatranscriptomic reads, and Luminex xTAG Gastrointestinal Pathogen Panel (Luminex) results (*p* < 0.001). The metatranscriptomic results correlated positively with Rotavirus (for both Traditional and Luminex methods, *p* < 0.001). The detection of Norovirus and Sapovirus by metatranscriptomics was significantly correlated (*p* < 0.001) with the Luminex results. Metagenomic and metatranscriptomic results did not correlate with the detection of Astrovirus using Traditional or Luminex methods.

### Protists

Protists were detected by both metagenomics and metatranscriptomics. However, the metatranscriptomic results had a much higher sensitivity for the detection of protists than metagenomics. Positive correlations between the detection of *Cryptosporidium* in metatranscriptomic data and laboratory data were highly significant (*p* < 0.001). No associations were observed between metagenomic data and laboratory results for *Cryptosporidium*. There was no correlation between the detection of *Giardia* using Traditional or Luminex methods, and detecting *Giardia* using metagenomics or metatranscriptomics.

### Bacterial pathogens

The identification of bacterial pathogens from sequencing data is challenging, as commensal organisms and pathogens can have extremely high levels of genomic similarity. Laboratory diagnostics tend to differentiate commensal and pathogenic organisms using genes or phenotypes associated with pathogenicity. Our results show that metagenomics and metatranscriptomics can both identify bacterial pathogens with differing sensitivities. For *Campylobacter*, positive correlations were observed between sequencing and all laboratory results (*p* < 0.001). *Salmonella* displayed positive correlations between sequencing data and both Traditional (*p* < 0.001) and Luminex (*p* < 0.25) diagnostics. *C. difficile* metatranscriptomic sequencing data positively correlated with both Traditional (*p* < 0.25) and Luminex (*p* < 0.001) diagnostics. *Y. enterocolitica* sequencing data positively correlated with Luminex results as follows: *C. difficile* metatranscriptomic reads (*p* < 0.001), *Y. enterocolitica* metatranscriptomic reads (*p* < 0.01), and *Y. enterocolitica* metagenomic reads (*p* < 0.001).

*E. coli* and *Shigella* are closely-related species; detection of *Shigella* in metagenomic data correlated positively with Traditional and Luminex diagnostics (*p* < 0.25), while *E. coli* showed a non-significant correlation (*p* > 0.25). *Vibrio cholerae* were not detected in either metagenomic or metatranscriptomic data, consistent with laboratory diagnostics, which identified no *V. cholerae* infections.

A summary of all correlations between the detection of GI pathogens in sequencing reads and laboratory data and their significance are provided in Additional Files 4, 6 and 7.

### Case-studies for the use of metatranscriptomics in pathogen surveillance

#### Complete genomes from diarrhoeal-associated Adenovirus can be detected in both metagenomic and metatranscriptomic data

**Fig. 3:**
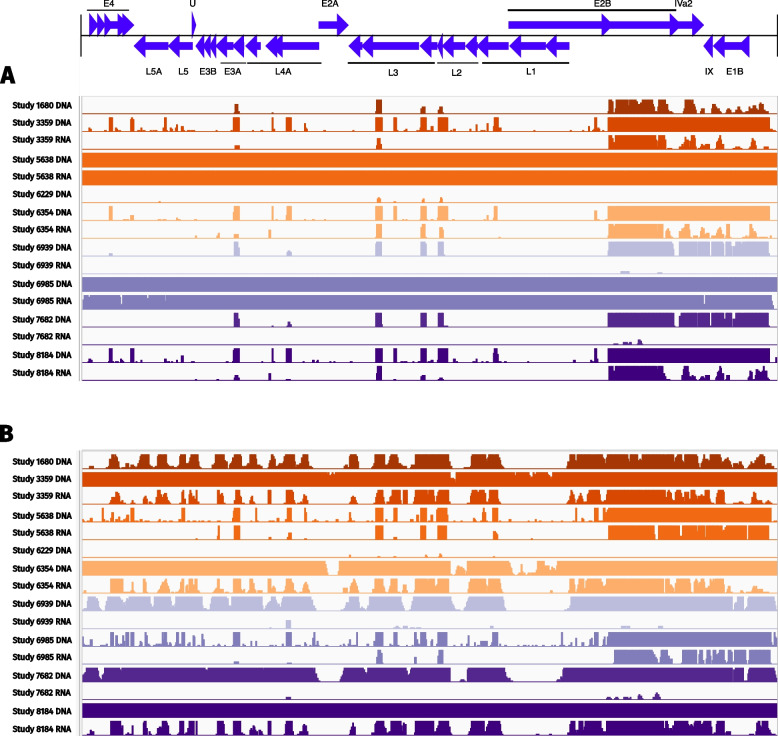
Adenovirus can be detected through its genomic material and the expression of transcript, directly from stool. Coverage graphs display the breadth of coverage (%) for both DNA and RNA across nine samples, chosen on the basis of positive results through gold-standard laboratory methods. Coverage values were generated via mapping to (A) human adenovirus F40 (NC_001454.1) and (B) F41 (DQ315364.2). For RNA, Study 1680 and 6229 are omitted from this visualisation due to insufficient coverage when mapping to F40. The x-axis shows genomic coordinates, while the y-axis indicates sequencing depth at each position. Colours group sequencing data by patient, with sample labels shown on the left-hand side

Whilst Adenovirus is a DNA virus, Adenovirus could also be detected in RNA-seq, reflecting the active role of Adenovirus in acute gastroenteritis. There was a strong correlation between the detection of Adenovirus species F in metagenomic and metatranscriptomic data, and detection using Luminex methods (see Additional File 4 and Fig. 2). In four out of nine samples, 5638 (99.9% coverage in DNA and RNA), 6985 (99.9% in DNA, 99.2% in RNA), 3359 (99.6% in DNA, 52.6% in RNA), and 8184 (99.8% in DNA, 56.6% in RNA), substantial genome coverage was achieved in both metagenomic and metatranscriptomic datasets. While DNA sequencing yielded more complete genomes, RNA sequencing still captured a broad representation of Adenovirus species F transcriptomes, sufficient to confirm their presence and subtype (see Fig. 3A for F40 and Fig. 3B for F41). The full mapping statistics for all samples are provided in Additional File 5. These results demonstrate the potential of metatranscriptomics to directly capture the virome from clinical samples, including DNA viruses relevant to the condition of interest.

### *Cryptosporidium*-associated RNA viruses facilitate detection directly from stool

Another interesting observation was the correlation (*p* < 0.001, see Fig. 2) between the detection of *Cryptosporidium* using metatranscriptomics and the detection of *Cryptosporidium* in the laboratory. In contrast, detecting *Cryptosporidium* using metagenomics did not correlate with laboratory results. Mapping revealed that *Cryptosporidium* was accurately identified in metatranscriptomic data due to the presence of *Cryptosporidium parvum* virus (CSpV1), which is a symbiotic RNA virus. CSpV1 was identified in 33 metatranscriptomic samples (Table 3). Of these 33 samples, nine received a positive result using Traditional methods, whilst 16 were positive by Luminex. CSpV1 received a high-confidence score (0.995) in 21 out of the 33 samples (see Additional File 4: Fig S2), with the percentage breadth of genome coverage ranging from 57.1 to 100%. However, CSpV1 detection was not significantly correlated with the laboratory detection of *Cryptosporidium* (see Additional Files 8 and 9), possibly due to lower prevalence of CSpV1 in these data. This contrasts with previous literature suggesting 100% prevalence in *C. parvum* [31, 32]). The findings suggest that CSpV1 could be a promising biomarker for human *Cryptosporidium* infection, though further validation is definitely needed.

**Table 3:** Identification of CSpV1 in metatranscriptomic data in comparison to results from *Cryptosporidium* laboratory diagnostics. For both Traditional and Luminex results, NA represents instances where a diagnostic test could not be performed

Study ID	Traditional result	Luminex result	TAXID	Read count	Coverage breadth (%)	Coverage depth (fold)	PanGIA score
238	0	1	675,060.1	24,334	0.9937	1057.6231	0.995
299	0	1	675,060.1	17,324	0.9907	742.9534	0.995
347	0	1	675,060.1	18,914	0.9513	820.9334	0.995
1530	NA	0	675,060.1	132,778	0.9997	5789.0705	0.995
1730	0	1	675,060.1	277,178	1	12,099.0436	0.995
1868	1	1	675,060.1	25,942	0.9836	1121.2385	0.995
1996	NA	0	675,060.1	490	0.9659	21.2555	0.995
2237	0	0	675,060.1	54	0.5705	2.3751	0.995
2270	1	1	675,060.1	2,481,486	1	108,131.0308	0.995
4580	0	0	675,060.1	136	0.8757	5.711	0.995
4667	1	1	675,060.1	45,590	1	1975.986	0.995
4922	1	1	675,060.1	11,154	0.9438	474.8819	0.995
5019	1	1	675,060.1	27,990	0.997	1230.6458	0.995
5195	0	0	675,060.1	526	0.8951	22.775	0.995
5215	1	1	675,060.1	43,408	0.9997	1895.0209	0.995
5563	0	1	675,060.1	85,048	0.9988	3700.396	0.995
5675	0	1	675,060.1	8338	0.9913	360.4002	0.995
6446	1	1	675,060.1	2200	0.9668	94.7941	0.995
6602	0	1	675,060.1	5770	0.9949	250.243	0.995
6912	1	1	675,060.1	21,542	0.8655	932.6551	0.995
7233	1	1	675,060.1	22,898	1	1005.3114	0.995
1817	NA	0	675,060.1	14	0.2624	0.601	0.601
1548	0	0	675,060.1	12	0.2585	0.535	0.535
1111	NA	0	675,060.1	10	0.2409	0.4471	0.4471
769	NA	0	675,060.1	10	0.2445	0.439	0.439
1436	NA	0	675,060.1	6	0.1491	0.2687	0.2687
54	0	0	675,060.1	6	0.1626	0.2469	0.2469
127	0	NA	675,060.1	4	0.0819	0.1793	0.1793
6890	NA	0	675,060.1	4	0.1136	0.179	0.179
5734	0	0	675,060.1	4	0.0562	0.1787	0.1787
360	0	0	675,060.1	4	0.1482	0.1781	0.1781
2279	0	0	675,060.1	4	0.0855	0.1614	0.1614
369	0	0	675,060.1	4	0.1384	0.1599	0.1599

### Generation of a complete transcriptomic profile for *Salmonella*

Metatranscriptomic analysis of stool from a patient with a laboratory-confirmed *Salmonella* infection yielded functional insights that cannot be achieved with Traditional and Luminex diagnostics. A high-quality transcriptomic profile was generated from 12.7 million sequence reads that mapped to the genome of *S. enterica* serovar Enteritidis PT4 strain P125109. A variety of environmentally responsive *Salmonella* genes were highly expressed (as defined by Kröger et al. 2013; *Cell Host Microbe* [33]), likely reflecting the physicochemical stresses the bacteria had been exposed to in the stool sample, during storage and/or transport. Examples include *ahpC* (oxidative stress), *hmpA* (nitrosative stress), *phoH* (phosphate starvation), *pspA* (extracytoplasmic stress) and the *rpoE* and *rpoS* transcription factor genes, as can be seen with the SalCom data visualisation tool (https://bioinf.gen.tcd.ie/cgi-bin/salcom.pl [33]). The unexpected discovery that the metatranscriptomic analysis of a raw, aged human stool sample can generate a comprehensive gene expression profile of a *Salmonella* pathogen, regardless of storage conditions, should be exploited in the future.

The *S.* Enteritidis transcripts from this novel gene expression data can be visualised and interrogated in a bespoke genome browser (https://s.hintonlab.com/study_74).

### Higher RNA/DNA ratios can distinguish negative and positive samples for *Campylobacter* and *C. difficile*

**Fig. 4:**
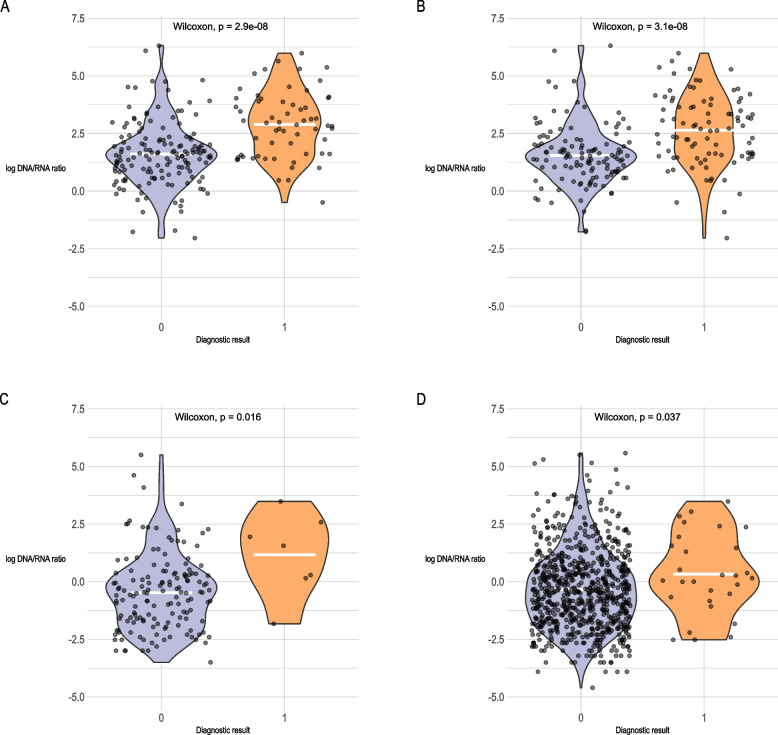
Higher RNA/DNA ratios were observed in samples that tested positive for *Campylobacter* and *C. difficile* by gold-standard diagnostics. Violin plots display the distribution of RNA/DNA ratios (logged for visualisation purposes) in samples classified as positive (0) or negative (1) based on gold-standard diagnostics. Panels correspond to the following pathogens and diagnostic tests: **A ***Campylobacter* Traditional, **B ***Campylobacter* Luminex, **C ***C. difficile* Traditional, **D ***C. difficile* Luminex. White lines indicate mean RNA/DNA ratios and *p*-values are the result of statistical comparisons between ratios in positive and negative samples, performed using the Wilcoxon rank-sum test

To demonstrate the complementary power of metagenomics and metatranscriptomics for clinical diagnostics and validate the relationship between pathogen presence and metatranscriptomic detection, we compared the ratios of mapped RNA and DNA reads (RNA/DNA) in positive and negative pathogen samples for *Campylobacter* and *C. difficile* (Additional File 10). These pathogens were selected due to their high infectious intestinal disease burden in community and hospital settings [34, 35], and their well-documented roles in diarrhoeal disease [36, 37]. Higher RNA/DNA ratios were observed in samples that tested positive for a pathogen via gold-standard diagnostics (Fig. 4). *Campylobacter* and *C. difficile* both displayed RNA/DNA ratios in samples that were significantly higher in samples that were positive via gold-standard diagnostics than in negative samples (see Fig. 4). As expected, there was no detection of *V. cholerae* in our metatranscriptomic data, which mirrors gold-standard diagnostics and reinforces the specificity of metatranscriptomics.

## Discussion

Here, we have shown that metagenomic and metatranscriptomic approaches provide agnostic detection of important UK GI pathogens from human stool. While multi-omics has been extensively explored in various biomedical applications [15, 17, 38], our study represents the largest systematic benchmarking of metagenomics and metatranscriptomics against existing diagnostic workflows for community-acquired GI infections. The primary impact of this work lies within GI pathogen diagnostics, where we have provided a large-scale evaluation of the performance of this multi-omic approach in comparison to gold-standard diagnostics. Our findings demonstrate the potential for improving current GI pathogen diagnostics as follows:

### Improvements within the scope of current diagnostics

Sequencing direct extracts from stool could minimise the time required for pathogen detection, allowing more laborious detection methods such as cultivation to be appropriately tailored to confirm the presence of the suspected pathogens.

The metatranscriptomic strategy displays increased sensitivity in comparison to metagenomics for *Campylobacter*, *C. difficile*,* Cryptosporidium* and *Giardia*, whilst metagenomics displayed increased sensitivity for other GI pathogens including Adenovirus, pathogenic *E. coli*, *Salmonella*, *Shigella* and *Y. enterocolitica*. Direct extraction of RNA from stool represents a single sample format and cultivation-independent process for detecting a broad range of GI pathogens, including unexpected aetiological agents and those that cannot be detected by metagenomic sequencing, such as RNA viruses. The observation of near-complete genome coverage for Human mastadenovirus F in both the metagenome and metatranscriptome highlights the potential to optimise metatranscriptomic sequencing from stool to capture the virome, including DNA virus transcriptomes relevant to clinical conditions. This finding is supported by previous clinical studies, which used metatranscriptomics to simultaneously measure the virome, microbiome, and host response [17]. Our data and previous studies [38] confirm the ability to characterise disease-related microbiomes with increased sensitivity via metatranscriptomics.

Increased sensitivity for the detection of protists of concern in GI infections was also demonstrated. Our visualisations of metagenomic and metatranscriptomic reads (Fig. 1) showed that metatranscriptomic data provide greater sensitivity for detecting *Cryptosporidium* and *Giardia* (protists). Additionally, our multivariable model demonstrated the strong correlation and high significance between the detection of *Cryptosporidium* in the laboratory and in metatranscriptomic data. This finding aligns with a previous study showing that metatranscriptomics can improve the sensitivity of parasite detection (e.g. *Plasmodium*)^27^ using other sample types. Notably, the aforementioned study detected 23% more blood infections when using metatranscriptomics over Traditional methods [39]. These data indicate that viruses associated with *Cryptosporidium*, like CSpV1, can improve the sensitivity of detection, especially using RNA-based methods. In contrast, metagenomic approaches for identifying *Cryptosporidium* showed inconsistency and lacked correlation with laboratory-confirmed cases, implying potential limitations in DNA-based detection Methods for this pathogen.

CSpV1 has recently been reported in various subtypes of *C. parvum* from diarrhoeic farm animals [32, 40], but it is not currently used as a diagnostic marker in humans. These results highlight the advantages of metatranscriptomics for *Cryptosporidium* surveillance, where the use of metagenomics alone could result in missed identification. This suggests that RNA viruses could be considered sensitive biomarkers for *Cryptosporidium* and other protists, though additional validation is needed. Finally, our results also suggest that higher RNA/DNA ratios may be indicative of a positive pathogen diagnosis. The relationship between RNA/DNA ratios and diagnostic accuracy is a result that requires further validation, but it is significant for those interested in adopting sequencing-based diagnostic methods. Overall, our findings reveal that RNA is a valuable diagnostic target for the detection of pathogens of low abundance that reduces false-positive signals from commensals. Our approach could influence the future allocation of resources for reference laboratory diagnostics.

### Bridging gaps not addressed by current diagnostics

Metatranscriptomic data could fill gaps in areas of clinical relevance that are not fulfilled by routine clinical diagnostics. Firstly, metagenomic and metatranscriptomic data permits the identification of multiple species and strains within a sample (Fig. 2) including novel pathogens. Such analysis is beyond the scope of our study, but has been used to successfully identify novel pathogens from the stools of various mammalian species [41, 42]. Additionally, we have demonstrated the ability to rapidly generate gene expression profiles for pathogens of concern, without prior enrichment. Finally, we have generated illuminating metatranscriptomic data from a human diarrhoeal sample. Future studies could generate true disease-state expression profiles by using appropriate methodology. From a clinical perspective, the use of metagenomic and metatranscriptomic sequencing has the potential to reveal the effects of interventions [43] and to accurately investigate host–pathogen dynamics during genuine human infections [17].

### Limitations

In certain scenarios, metagenomic sequencing captures more information than metatranscriptomic sequencing. For DNA viruses, while it is possible to capture expression profiles, optimisation is needed to improve this process. Our data demonstrate that key biological insights can be obtained, but further refinement is necessary to generate robust RNA-seq data for additional pathogens. For example, *E. histolytica* was not captured by metagenomic or metatranscriptomic approaches, a finding that requires further investigation. These discrepancies highlight the challenges of relying on molecular assays alone for pathogen detection. The inconsistencies observed with *E. histolytica* in the Luminex xTAG GPP assay suggest potential false positives, aligning with previous studies that have reported similar issues [44, 45]. In contrast, metagenomic sequencing may offer a more accurate representation of pathogen presence, though its sensitivity is still influenced by sample storage conditions and methodological constraints. Further optimisation of sequencing protocols, particularly in relation to nucleic acid extraction and reference database curation, is necessary to enhance the detection of protozoan pathogens and minimise diagnostic discrepancies.

Future adaptation of our workflow is needed for the accurate identification of *E. coli* pathovariants from sequencing data. *Shigella* and *E. coli* pathovariants are extremely similar on a genome-wide (and taxonomic) level [46], and are currently distinguished using specific gene-based assays [47]. In contrast, our study drew correlations between pathogens in reads and laboratory tests based on taxonomy. As our correlation was taxonomy-based, and *E. coli* reads were present in all stool samples, it was not possible to associate the presence of *E. coli with* the gene-based assays used for *E. coli* pathovariant identification. The high genomic similarity between *E. coli* and *Shigella* may explain the limited overlap between *Shigella* sequencing reads and laboratory tests (Fig. 2). However, our findings indicate that a fraction of *k*-mers (see Fig. 2, as well as Additional Files 4, 6 and 7) did distinguish *E. coli* from *Shigella* sequencing reads, demonstrating that *k*-mer-based approaches hold promise. Methods which leverage variable-length *k*-mer comparisons [48, 49] to distinguish bacterial isolates based on shared sequence divergence and gene content could be trialled in future iterations of our workflow to improve resolution. Future work should also validate our approach on a range of sample types (beyond stool) to ensure robustness and reliability.

Metagenomic and metatranscriptomic sequencing have the potential for clinical diagnostics but face challenges in routine use due to high costs [50], infrastructure needs and a shortage of skilled personnel [51]. Cloud-based analysis, decentralised sequencing platforms like Oxford Nanopore, and sample automation are being explored to overcome these challenges [52, 53].

These approaches are currently being integrated into biosecurity frameworks for emerging pandemics [54], used for antimicrobial stewardship to reduce the duration of hospitalisation [55] and prioritised in national public health strategies [56], highlighting their increasing importance.

Standardised protocols and validated controls are crucial for ensuring reproducibility [7], supported by guidelines that assist pathology labs in achieving regulatory compliance (UKAS and ISO 15189:2012 accreditation [57]). The application of microbial reference materials (e.g. ZymoBIOMICS Microbial Community Standards) can be beneficial [7], and quality control practices, such as validating samples through external accreditation programs (e.g. Quality Control for Molecular Diagnostics (QCMD)) are viable approaches to achieve clinical reliability [55].

Ultimately, even with these complexities in mind, the total cost of care and treatment often exceeds the fatal cost of a missed or inaccurate initial diagnosis [58]. Our study represents an important step toward clinical application, though further refinements will be needed before full deployment.

## Conclusions

With sufficient benchmarking, the diagnosis of various GI pathogens can be confidently achieved through the direct sequencing of clinical samples. We have demonstrated that metatranscriptomics can detect active DNA viruses and enhance sensitivity for protists by using RNA viruses as biomarkers. Perhaps the value of clinical metagenomics has been overstated, and metatranscriptomics could offer a comprehensive approach to both detect disease-relevant pathogens and understand their biology.

To our knowledge, this study is the first to demonstrate and quantify the potential advantages of metatranscriptomics for gastrointestinal surveillance in the UK by direct comparison with validated diagnostics of all major community pathogens. Even in samples that lacked RNA-stabilisation, we report that metatranscriptomics offers improved sensitivity over metagenomics and expands the range of organisms detectable via sequencing of nucleic acids. This work provides a foundation for advancing metatranscriptomics as a diagnostic tool in clinical settings.

## Supplementary Information

Additional file 1: Detailed description of Traditional laboratory methods used in the INTEGRATE study (companion to Table 2).

Additional file 2: Diagnostic results from Traditional methods and Luminex assays in the INTEGRATE study (companion to Table 1).

Additional file 3: BIOM file containing all k-mer counts and metadata for the samples in this study.

Additional file 4: Fig S1: Complete overview of correlations observed between sequencing data and laboratory tests for major GI community pathogens in the United Kingdom. In the heatmap, the darker the quadrant, the stronger the correlation (coefficient) between pathogen detection in sequencing data (metagenomic or metatranscriptomic) and laboratory results (Luminex or Traditional methods). Blue represents a positive correlation, while red indicates a negative correlation. Asterisks in quadrants indicate the statistical significance of correlations as follows: *: *p* < 0.25; **: *p *< 0.05; ***: *p* < 0.01; ****: *p* < 0.001. Black quadrants represent where no correlation between pathogen detection in sequencing data and laboratory results was identified. No statistically significant correlation was found between the sequencing and diagnostic test for Astrovirus, *E. histolytica*, *Giardia* or *V. cholerae*. Fig S2: Concordance of CSpV1 detection with *Cryptosporidium *diagnoses. This figure compares the detection of CSpV1 using mapping- and k-mer-based approaches alongside diagnostic results for *Cryptosporidium*. It complements Figure 1 by visualising concordance between Traditional laboratory methods, metagenomics, and metatranscriptomics. Panels show: (A) Traditional diagnostic results vs. metagenomics, (B) Traditional results vs. metatranscriptomics, (C) Luminex results vs. metagenomics, and (D) Luminex results vs. metatranscriptomics.

Additional file 5: Coverage statistics for Adenovirus F40 and F41.

Additional file 6: Correlation coefficients between k-mers and clinical metadata, generated using MaAsLin2.

Additional file 7: Significance levels for correlation coefficients between k-mers and clinical metadata, produced via MaAsLin2.

Additional file 8: Correlation coefficients between reads mapped to *Cryptosporidium parvum* virus 1 and clinical metadata, produced via MaAsLin2.

Additional file 9: Significance levels for correlations between reads mapped to *Cryptosporidium parvum* virus 1 and clinical metadata, produced via MaAsLin2.

Additional file 10: RNA/DNA ratios and their correspondence to positive and negative results based on gold standard diagnostics.

## Data Availability

Illumina sequence reads for metagenomic and metatranscriptomic sequencing experiments with human data removed have been deposited in the European Nucleotide Archive (ENA) under ENA project accession number PRJEB62473. Code necessary to reproduce these analyses are available on GitHub [59].
