# Supplementary material for: Enhancing infectious intestinal disease diagnosis through metagenomic and metatranscriptomic sequencing of 1000 human diarrhoeal samples
Source: Genome Med. 2025 May 20;17:55. doi: 10.1186/s13073-025-01478-w (PMC12090668; doi:10.1186/s13073-025-01478-w)
Supplement: Supplementary file 4 — Additional file 4: Fig S1: Complete overview of correlations observed between sequencing data and laboratory tests for major GI community pathogens in the United Kingdom. In the heatmap, the darker the quadrant, the stronger the correlation (coefficient) between pathogen detection in sequencing data (metagenomic or metatranscriptomic) and laboratory results (Luminex or Traditional methods). Blue represents a positive correlation, while red indicates a negative correlation. Asterisks in quadrants indicate the statistical significance of correlations as follows: *: p < 0.25; **: p < 0.05; ***: p < 0.01; ****: p < 0.001. Black quadrants represent where no correlation between pathogen detection in sequencing data and laboratory results was identified. No statistically significant correlation was found between the sequencing and diagnostic test for Astrovirus, E. histolytica, Giardia or V. cholerae. Fig S2: Concordance of CSpV1 detection with Cryptosporidium diagnoses. This figure compares the detection of CSpV1 using mapping- and k-mer-based approaches alongside diagnostic results for Cryptosporidium. It complements Figure 1 by visualising concordance between Traditional laboratory methods, metagenomics, and metatranscriptomics. Panels show: (A) Traditional diagnostic results vs. metagenomics, (B) Traditional results vs. metatranscriptomics, (C) Luminex results vs. metagenomics, and (D) Luminex results vs. metatranscriptomics. [file 13073_2025_1478_MOESM4_ESM.pdf]

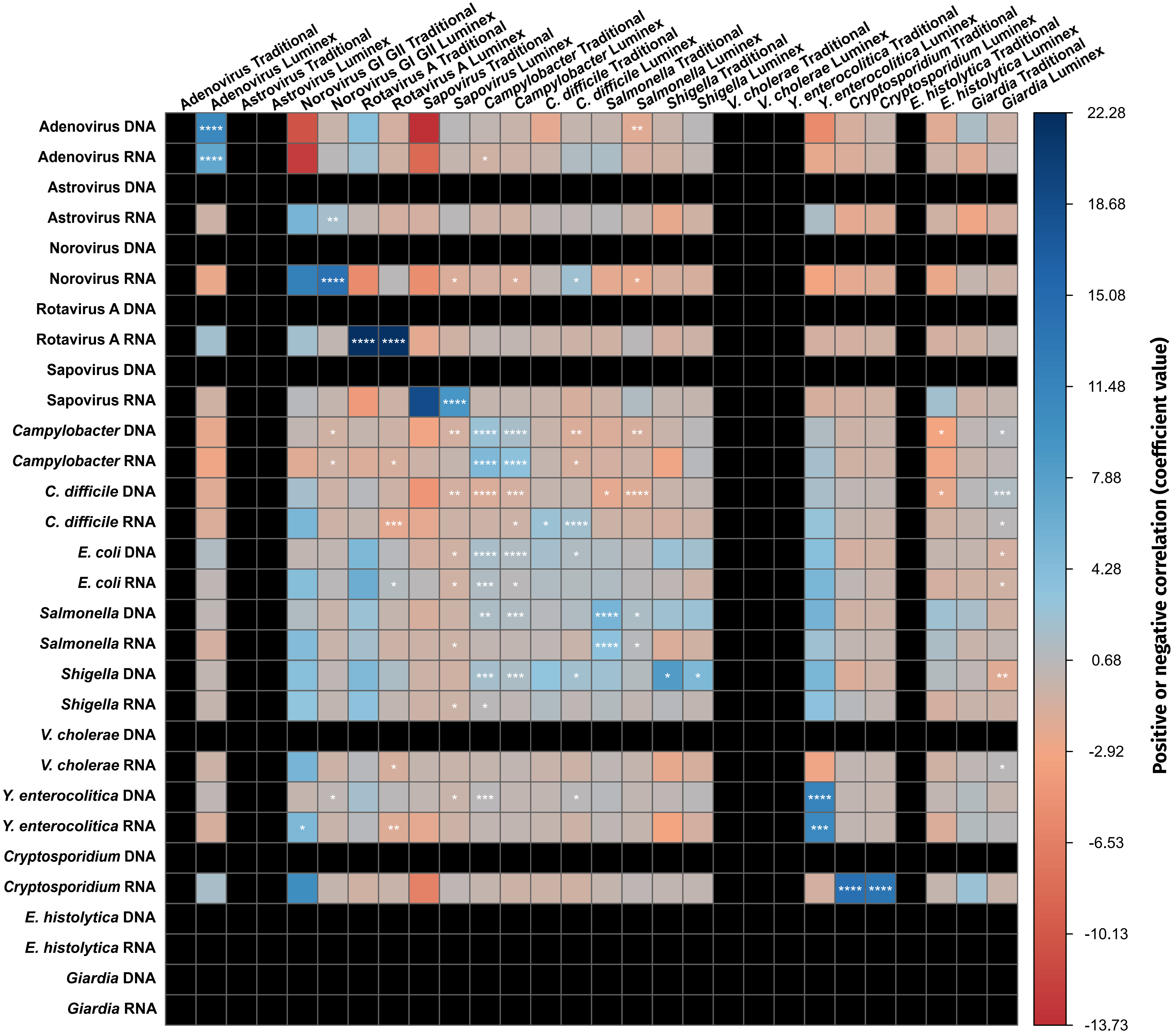

Key for significance values:
*** = <0.25, ** = <0.05, *** = <0.01, **** = <0.001**

Fig S1: Complete overview of correlations observed between sequencing data and laboratory tests for major GI community pathogens in the United Kingdom.

In the heatmap, the darker the quadrant, the stronger the correlation (coefficient) between pathogen detection in sequencing data (metagenomic or metatranscriptomic) and laboratory results (Luminex or Traditional methods). Blue represents a positive correlation, while red indicates a negative correlation. Asterisks in quadrants indicate the statistical significance of correlations as follows: *: $p < 0.25$; **: $p < 0.05$; ***: $p < 0.01$; ****: $p < 0.001$. Black quadrants represent where no correlation between pathogen detection in sequencing data and laboratory results was identified.No statistically significant correlation was found between the sequencing and diagnostic test for Astrovirus, *E. histolytica* ,*Giardia* or *V. cholerae*.

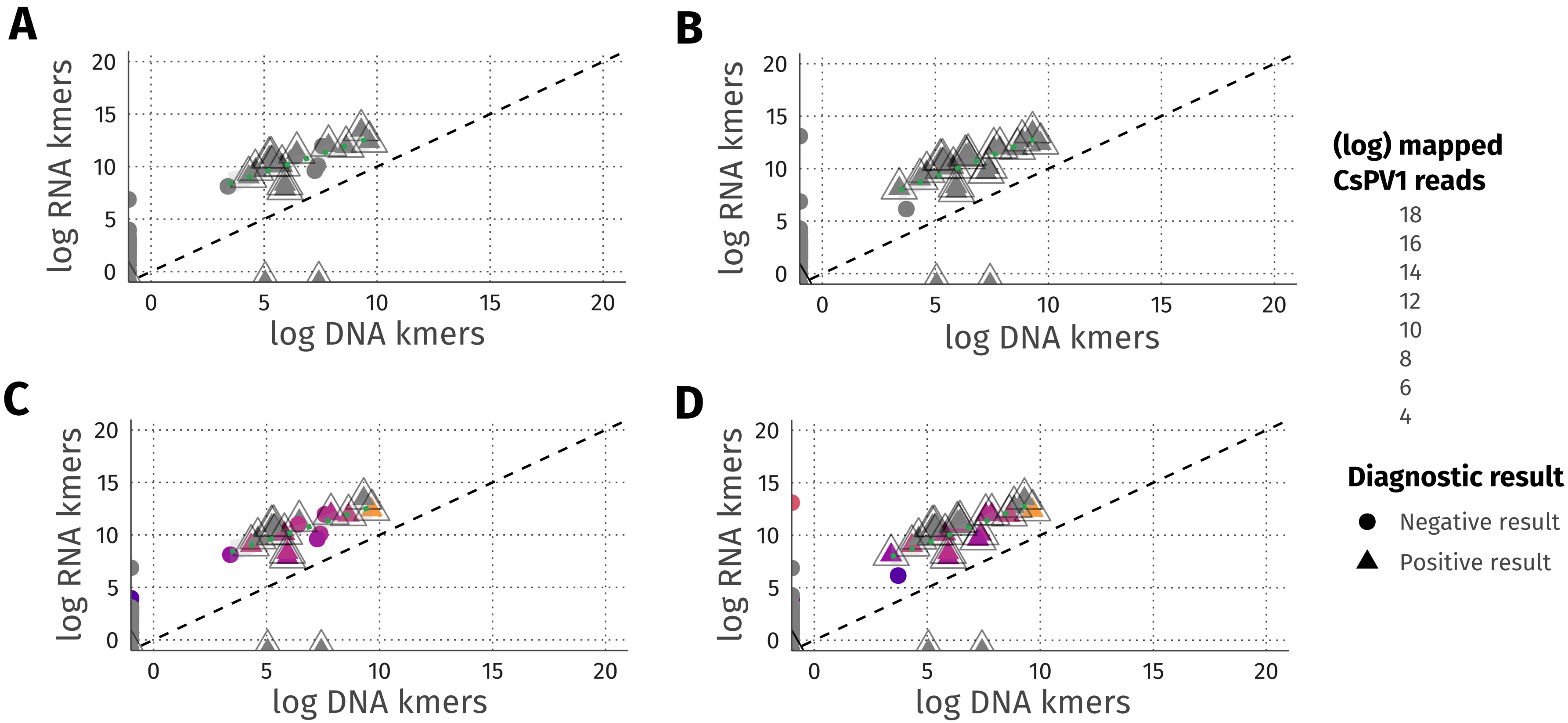

Fig S2: Concordance of CSpV1 detection with *Cryptosporidium* diagnoses.

This figure compares the detection of CSpV1 using mapping- and k-mer-based approaches alongside diagnostic results for *Cryptosporidium*. It complements Table 3 by visualising the concordance between Traditional laboratory methods, metagenomics, and metatranscriptomics using mapped reads. Panels show: (A) Traditional diagnostic results vs. metagenomics, (B) Traditional results vs. metatranscriptomics, (C) Luminex results vs. metagenomics, and (D) Luminex results vs. metatranscriptomics.
